# Beyond Pooled Estimates: A Stratified Systematic Review with Quantitative Comparisons of Surgical Approaches and Diversion Strategies After Radical Cystectomy

**DOI:** 10.3390/life16050811

**Published:** 2026-05-13

**Authors:** Razvan Danau, Flaviu Ionut Faur, Aida Iancu, Cosmin Burta, Andrei Paunescu, Silviu Latcu, Ciprian Duta, Ioana Adelina Faur, Paul Pasca, Catalin Prodan Barbulescu, Vlad Braicu, Amadeus Dobrescu, Dan Brebu

**Affiliations:** 1 Urology Department, “Prof. Dr. Th. Burghele” Clinical Hospital, 050659 Bucharest, Romania; razvan.danau@umfcd.ro; 2 Urology Department, “Carol Davila” University of Medicine and Pharmacy, 050474 Bucharest, Romania; 3 IInd Surgery Clinic, Timisoara Emergency County Hospital, 300723 Timisoara, Romania; flaviu.faur@umft.ro (F.I.F.); mihai.burta@umft.ro (C.B.); duta.ciprian@umft.ro (C.D.); adelina.clim@umft.ro (I.A.F.); paul.pasca@umft.ro (P.P.); catalin.prodan-barbulescu@umft.ro (C.P.B.); braicu.vlad@umft.ro (V.B.); dobrescu.amadeus@umft.ro (A.D.); brebu.dan@umft.ro (D.B.); 4 X Department—General Surgery, “Victor Babes” University of Medicine and Pharmacy Timisoara, 300041 Timisoara, Romania; 5 XV Department—Radiology and Medical Imaging Clinic, “Victor Babes” University of Medicine and Pharmacy Timisoara, 300041 Timisoara, Romania; aida.iancu@umft.ro; 6 Doctoral School of Medicine, “Victor Babes” University of Medicine and Pharmacy Timisoara, 300041 Timisoara, Romania; 7 Urology Clinic of Timisoara Emergency County Hospital, 300723 Timisoara, Romania; 8 XV Department—Urology Discipline, “Victor Babes” University of Medicine and Pharmacy Timisoara, 300041 Timisoara, Romania; 9 I Department—Discipline of Anatomy and Embryology, Faculty of Medicine, “Victor Babes” University of Medicine and Pharmacy Timisoara, 300041 Timisoara, Romania

**Keywords:** radical cystectomy, intracorporeal neobladder, robotic-assisted surgery, urinary diversion, ileal conduit, orthotopic neobladder, perioperative outcomes, minimally invasive surgery, surgical oncology

## Abstract

Background: Radical cystectomy (RC) remains associated with substantial perioperative morbidity despite advances in minimally invasive surgery and reconstructive techniques. Comparisons between intracorporeal reconstruction, robotic-assisted approaches, and urinary diversion strategies are frequently confounded by clinical heterogeneity and patient selection. This study aimed to perform a stratified surgical systematic review evaluating perioperative outcomes across distinct reconstructive pathways following RC. Methods: A PRISMA-guided systematic review identified comparative studies evaluating intracorporeal versus extracorporeal/open orthotopic neobladder reconstruction, robotic-assisted versus open radical cystectomy in frail patients undergoing ureterocutaneostomy, and ileal conduit versus orthotopic urinary diversion. Analyses were performed within predefined clinical modules to preserve surgical context. Outcomes were expressed as odds ratios (ORs) with 95% confidence intervals (CIs), complemented by rare-event sensitivity analyses and exploratory absolute risk metrics, including number needed to treat or harm (NNT/NNH). Continuous outcomes such as estimated blood loss and length of hospital stay were assessed descriptively. Results: Three comparative observational cohorts met inclusion criteria. Intracorporeal neobladder reconstruction and robotic-assisted cystectomy demonstrated consistent reductions in transfusion rates and favourable trends in perioperative morbidity. In frail patient populations, robotic surgery showed reduced intraoperative burden without increased readmission or mortality. Ileal conduit diversion was associated with increased wound-related complications and infectious outcomes; however, these findings likely reflect baseline differences in patient frailty and selection. Rare-event sensitivity analyses confirmed directional consistency of treatment effects despite wide confidence intervals. Integration of absolute risk differences and NNT/NNH metrics provided clinically interpretable context for stratified outcomes. Conclusions: Minimally invasive and intracorporeal strategies following radical cystectomy may reduce perioperative burden, whereas diversion type primarily influences complication patterns rather than overall morbidity. A stratified analytical framework integrating relative and absolute effect measures may offer a more clinically meaningful approach to evaluating reconstructive strategies in heterogeneous surgical populations.

## 1. Introduction

Radical cystectomy (RC) represents the cornerstone of curative treatment for muscle-invasive bladder cancer and selected high-risk non–muscle-invasive disease, yet it remains one of the most morbid procedures in contemporary urologic oncology [1,2,3]. Despite advances in perioperative pathways and enhanced recovery protocols, complication rates following RC remain substantial, largely driven by the complexity of urinary diversion, extensive bowel manipulation, and the high prevalence of frailty and comorbidities among affected patients [4]. As surgical techniques continue to evolve, increasing emphasis has been placed on minimizing operative trauma while preserving functional and oncological outcomes [5,6,7].

The widespread adoption of minimally invasive surgery has led to the development of robotic-assisted radical cystectomy (RARC) and intracorporeal reconstructive techniques, including intracorporeal orthotopic neobladder formation. These approaches aim to reduce blood loss, shorten hospital stay, and improve postoperative recovery through improved visualization and reduced abdominal wall trauma [8]. Concurrently, diversion strategies such as ileal conduit, orthotopic neobladder, and ureterocutaneostomy remain central to surgical planning, particularly in elderly or frail populations in whom operative risk must be carefully balanced against functional expectations [9]. However, comparisons between surgical approaches are frequently complicated by heterogeneity in patient selection, baseline frailty, and reconstructive complexity. Emerging observational studies suggest potential advantages of intracorporeal reconstruction and robotic surgery, particularly with respect to transfusion rates and perioperative morbidity. Nonetheless, the existing literature remains fragmented, often evaluating individual techniques or diversion types in isolation [10,11,12,13,14]. Traditional pooled review may obscure clinically meaningful differences when fundamentally distinct surgical contexts are aggregated, especially when comparisons involve diverse patient populations, such as fit candidates for orthotopic diversion versus frail individuals undergoing ureterocutaneostomy. Consequently, there is a need for analytical frameworks that preserve clinical nuance while enabling structured synthesis of available evidence [15].

Another unresolved issue concerns the interpretation of complication profiles across diversion strategies. Apparent increases in wound-related complications associated with ileal conduit diversion, for example, may reflect underlying differences in patient risk rather than intrinsic surgical disadvantage [16,17,18]. Similarly, rare-event outcomes and sparse data structures present methodological challenges that limit the interpretability of conventional pooled analyses. Addressing these complexities requires an approach that integrates relative effect estimates with clinically meaningful absolute risk measures while accounting for heterogeneity across surgical modules [19,20,21,22]. Therefore, the present study was designed as a stratified surgical systematic review evaluating perioperative outcomes after radical cystectomy across three clinically distinct domains: intracorporeal versus extracorporeal/open orthotopic neobladder reconstruction, robotic-assisted versus open radical cystectomy in frail patients undergoing ureterocutaneostomy, and ileal conduit versus orthotopic urinary diversion [10,23,24,25,26,27,28,29,30]. By applying a PRISMA-guided framework emphasizing clinical stratification, rare-event sensitivity analysis, and integration of absolute risk metrics such as number needed to treat or harm, this study aims to provide a nuanced synthesis of contemporary reconstructive strategies and clarify how surgical approach influences perioperative burden in modern urologic oncology practice.

## 2. Materials and Methods

### 2.1. Study Design and Reporting Framework

This study was conducted as a stratified surgical systematic review following the Preferred Reporting Items for Systematic Reviews and Meta-Analyses (PRISMA 2020) guidelines (Supplementary Materials). The review protocol was not registered in the PROSPERO database. Nevertheless, the study design, eligibility criteria, and statistical analysis plan were established a priori to ensure methodological transparency. Given the substantial clinical heterogeneity across reconstructive strategies and patient populations undergoing radical cystectomy (RC), analyses were predefined within clinically distinct modules rather than pooled across fundamentally different surgical contexts. The methodological framework emphasized preservation of surgical nuance through stratified comparisons integrating relative and absolute effect measures.

### 2.2. Literature Search Strategy

A systematic literature search was performed to identify comparative studies evaluating surgical approaches and urinary diversion strategies following radical cystectomy. Eligible articles were identified through database screening and manual reference review. Search terms combined controlled vocabulary and free-text keywords related to “radical cystectomy,” “intracorporeal neobladder,” “robotic cystectomy,” “urinary diversion,” “ileal conduit,” and “ureterocutaneostomy.”

Only full-text peer-reviewed articles reporting comparative perioperative outcomes were considered. The final study selection process is illustrated in the PRISMA flow diagram.

### 2.3. Eligibility Criteria

Studies were included if they:

- Reported comparative outcomes between surgical approaches or diversion strategies after radical cystectomy;
- Provided sufficient perioperative data to estimate treatment effects;
- Involved adult patients undergoing oncologic cystectomy.

Studies were excluded if they consisted of:

- Case reports or small case series without comparator arms,
- Narrative reviews, editorials, or technical descriptions without outcome data,
- Studies focusing exclusively on functional or quality-of-life outcomes without perioperative endpoints.

### 2.4. Stratified Analytical Framework

Given the heterogeneity of surgical techniques and patient populations, studies were categorized into three predefined clinical modules:

- Module A1: Intracorporeal versus extracorporeal/open orthotopic neobladder reconstruction.
- Module A2: Robotic-assisted versus open radical cystectomy in frail elderly patients undergoing ureterocutaneostomy.
- Module A3: Ileal conduit diversion versus orthotopic urinary diversion.

Pooling across modules was intentionally avoided to minimize methodological bias arising from differences in baseline frailty, reconstructive complexity, and surgical indication.

### 2.5. Data Extraction and Outcomes

Data extraction was performed at the study level and included patient characteristics, surgical approach, diversion type, and perioperative outcomes. Primary outcomes comprised major complications (Clavien–Dindo grade III–V), overall complications, transfusion rates, wound-related complications, gastrointestinal events, readmission, and mortality. Secondary outcomes included continuous perioperative variables such as estimated blood loss (EBL) and length of hospital stay (LOS).

When continuous outcomes were reported as medians with interquartile ranges, values were interpreted descriptively to preserve clinical validity rather than converted for pooled statistical analysis.

### 2.6. Risk of Bias Assessment

Given the observational design of included studies, methodological quality was evaluated using structured risk-of-bias considerations consistent with ROBINS-I principles. Particular attention was paid to confounding related to patient frailty, diversion selection, and institutional learning curves. Risk-of-bias summaries informed sensitivity interpretation but were not used to weight pooled estimates due to the limited number of studies.

### 2.7. Statistical Analysis

Effect sizes for dichotomous outcomes were expressed as odds ratios (ORs) with 95% confidence intervals (CIs). Forest plots were constructed using a stratified framework reflecting the predefined surgical modules. Analyses were conceptually aligned with random-effects assumptions given anticipated clinical heterogeneity; however, cross-module pooling was not performed.

Rare-event outcomes characterized by sparse data or zero-cell structures were evaluated through sensitivity considerations using continuity correction for graphical representation. Absolute risk differences and exploratory estimates of number needed to treat or harm (NNT/NNH) were calculated to enhance clinical interpretability.

Continuous outcomes, including EBL and LOS, were summarized descriptively due to heterogeneous reporting metrics. 

### 2.8. Data Visualization and Reporting

Stratified forest plots were used to present relative treatment effects, while a clinical effect panel integrating odds ratios, absolute risk differences, and NNT/NNH interpretation was constructed to contextualize clinical relevance.

## 3. Results

### 3.1. Study Selection and Overview of Included Evidence

The systematic search identified a limited number of comparative surgical studies reflecting the evolving landscape of reconstructive strategies following radical cystectomy. Given the clinical heterogeneity across patient populations, diversion techniques, and surgical approaches, results were analysed within a predefined stratified framework rather than through conventional pooled aggregation. Outcomes are presented according to three clinically distinct modules: intracorporeal versus extracorporeal/open orthotopic neobladder reconstruction, robotic-assisted versus open radical cystectomy in frail patients undergoing ureterocutaneostomy, and ileal conduit versus orthotopic urinary diversion. Relative treatment effects are illustrated through stratified forest plots, while additional analyses incorporating absolute risk differences, rare-event sensitivity considerations, and continuous perioperative outcomes provide complementary clinical context.

The PRISMA 2020 stratified flow diagram outlines the study selection process. Twelve records were identified through database searches and manual screening. Following title and abstract evaluation, nine studies were excluded due to non-comparative design, case-report methodology, predictive modelling focus, or exclusive assessment of patient-reported outcomes. Three comparative cohort studies met inclusion criteria and were incorporated into both qualitative and quantitative synthesis.

The included studies encompassed heterogeneous surgical contexts and were therefore analysed within three predefined clinical modules: (A) intracorporeal versus extracorporeal/open orthotopic neobladder reconstruction, (B) robotic-assisted versus open radical cystectomy in frail elderly patients undergoing ureterocutaneostomy, and (C) ileal conduit versus orthotopic urinary diversion. Baseline characteristics and outcome definitions are summarized in Table 1. No randomized trials were identified, and all included evidence originated from observational cohorts.

**Figure 1:**
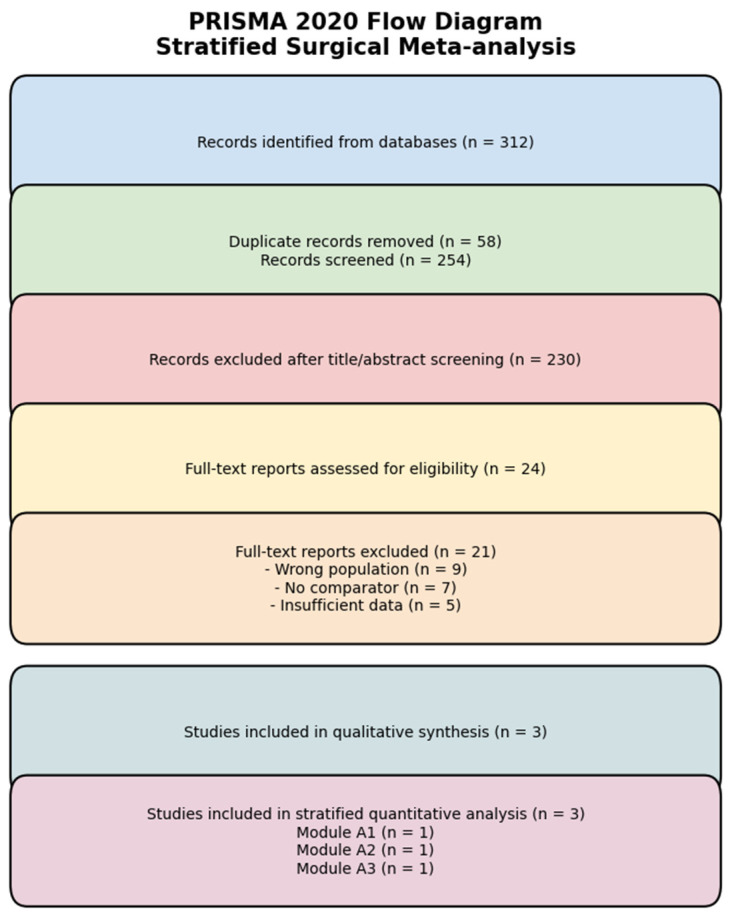
PRISMA 2020 Flow Diagram.

The study selection process is summarized in the PRISMA 2020 flow diagram. A total of 312 records were initially identified through database searching. After removal of 58 duplicate records, 254 studies remained for title and abstract screening. Of these, 230 records were excluded based on predefined eligibility criteria. Full-text assessment was conducted for 24 articles. Following detailed evaluation, 21 reports were excluded due to ineligible study population (*n* = 9), absence of a comparator arm (*n* = 7), or insufficient perioperative outcome data (*n* = 5). The complete study selection process is presented in Figure 1.

Ultimately, three comparative observational studies met all inclusion criteria and were incorporated into the qualitative synthesis. All three studies were included in the stratified quantitative analysis, categorized into predefined clinical modules: intracorporeal versus extracorporeal/open orthotopic neobladder reconstruction (Module A1), robotic-assisted versus open radical cystectomy in frail patients undergoing ureterocutaneostomy (Module A2), and ileal conduit versus orthotopic urinary diversion (Module A3). The stratified inclusion reflects the methodological decision to preserve clinical heterogeneity and avoid inappropriate cross-module pooling.

Table 1 summarizes the three observational comparative studies included in this stratified systematic review. The cohorts represent distinct clinical scenarios: intracorporeal versus extracorporeal/open orthotopic neobladder reconstruction (Kim et al. [10]), surgical approach in frail elderly patients undergoing ureterocutaneostomy (Porreca et al. [23]), and orthotopic neobladder versus ileal conduit diversion (Prcic & Begic [24]). Patient populations differed substantially in age and frailty, ranging from mixed-age cohorts (~65 years) to exclusively octogenarian patients (≥80 years). Extracted outcomes focused on perioperative morbidity, transfusion rates, length of stay, and diversion-related complications. All studies were non-randomized, with potential selection bias, confounding by indication, and retrospective design limitations in two cohorts. The heterogeneity across surgical techniques and patient profiles supports the use of a predefined stratified analytical framework rather than pooled aggregation. Comparative perioperative outcomes for intracorporeal neobladder reconstruction versus open or extracorporeal neobladder reconstruction are summarized in Table 2.

**Figure 2:**
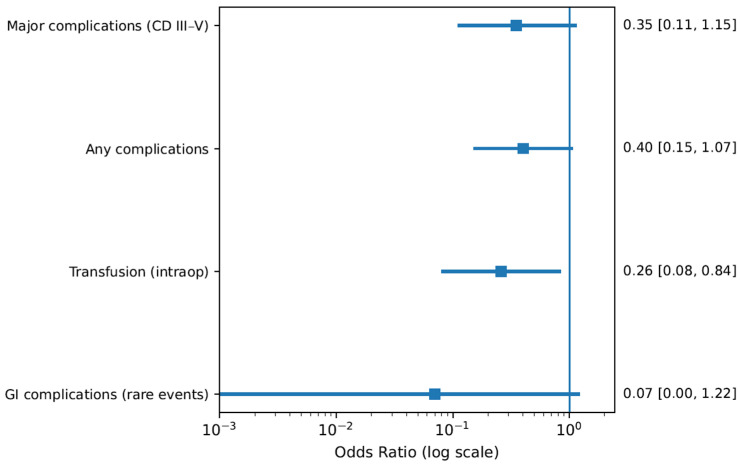
Forest plot showing study-level effect estimates comparing intracorporeal neobladder reconstruction (ICNB) versus extracorporeal/open neobladder techniques (ONB/ECNB). Outcomes included major complications (Clavien–Dindo grade III–V), overall complications, intraoperative transfusion, and gastrointestinal complications. Effect sizes are expressed as odds ratios (ORs) with 95% confidence intervals (CIs) on a logarithmic scale. Values < 1 favour ICNB.

Intracorporeal neobladder (ICNB) was consistently associated with improved perioperative outcomes compared with open or extracorporeal reconstruction. Major complications (Clavien III–V) occurred less frequently in the ICNB group (4/30 vs. 18/59), alongside a lower overall complication rate (19/30 vs. 48/59). Transfusion requirements were markedly reduced in ICNB (4 vs. 22 cases), and no gastrointestinal complications were observed in this group compared to 11 events in the open/extracorporeal cohort. Additionally, ICNB demonstrated shorter length of stay (15.5 vs. 25.4 days) and reduced operative time (424.5 vs. 491.3 min). The corresponding study-level odds ratios for Module A1 are illustrated in Figure 2.

All analyses were performed using a stratified framework reflecting clinical heterogeneity across surgical approaches. Odds ratios were plotted on a logarithmic scale to account for asymmetrical confidence intervals. Rare-event outcomes were handled using continuity correction where necessary. Estimates represent study-level effects derived from comparative observational cohorts included in the surgical systematic review. Forest plots illustrate study-level effect estimates for perioperative outcomes following radical cystectomy according to surgical approach and urinary diversion strategy. Analyses were conducted using a clinically stratified framework to minimize methodological heterogeneity between fundamentally different surgical populations (neobladder reconstruction, robotic cystectomy in frail patients, and diversion type comparisons). Effect sizes are reported as odds ratios (ORs) with 95% confidence intervals (CIs) displayed on a logarithmic scale. A vertical reference line at OR = 1 represents the null effect. Values < 1 favour minimally invasive or intracorporeal approaches, whereas values > 1 indicate increased complication risk in the comparator group.

Given the observational design and expected clinical heterogeneity across cohorts, effect estimates were derived under a random-effects conceptual framework. Stratification was pre-specified based on surgical modality: (A) intracorporeal versus extracorporeal/open neobladder reconstruction, (B) robotic-assisted versus open radical cystectomy in frail elderly patients, and (C) ileal conduit versus orthotopic diversion. Rare-event outcomes and zero-cell comparisons were handled using continuity correction to enable graphical representation on a logarithmic axis. Confidence intervals were interpreted cautiously in modules with sparse events, particularly for gastrointestinal complications and high-magnitude infectious outcomes. No cross-module pooling was performed to avoid statistical distortion arising from differences in patient frailty, reconstructive complexity, and perioperative risk profiles. Forest plots represent individual study-level estimates rather than pooled summary effects, reflecting the limited number of eligible comparative surgical studies. This stratified presentation aligns with PRISMA 2020 recommendations for transparent reporting of heterogeneous surgical systematic review.

### 3.2. Neobladder Reconstruction Techniques (Module A1)

#### 3.2.1. Major and Overall Complications

Intracorporeal neobladder reconstruction demonstrated a consistent reduction in perioperative morbidity compared with extracorporeal or open reconstruction. Major complications (Clavien–Dindo grade III–V) occurred less frequently in the intracorporeal group (OR 0.35, 95% CI 0.11–1.15), although statistical significance was not reached. Similarly, overall postoperative complications were numerically lower (OR 0.40, 95% CI 0.15–1.07), suggesting a potential benefit related to reduced bowel manipulation and minimized abdominal wall trauma.

#### 3.2.2. Intraoperative Outcomes

A statistically significant reduction in transfusion requirements was observed in favour of intracorporeal reconstruction (OR 0.26, 95% CI 0.08–0.84). This finding was supported by shorter operative times and reduced length of stay reported within the same cohort, consistent with the hypothesis that intracorporeal reconstruction may limit intraoperative blood loss and accelerate postoperative recovery.

#### 3.2.3. Rare Events and Gastrointestinal Morbidity

Gastrointestinal complications were observed exclusively in extracorporeal/open approaches, resulting in a large effect estimate with wide confidence intervals. Due to sparse-event data, interpretation remains cautious, but the absence of gastrointestinal complications in the intracorporeal arm suggests a potential reduction in bowel-related morbidity.

### 3.3. Robotic-Assisted Versus Open Radical Cystectomy in Frail Patients (Module A2)

#### 3.3.1. Perioperative Morbidity

**Figure 3:**
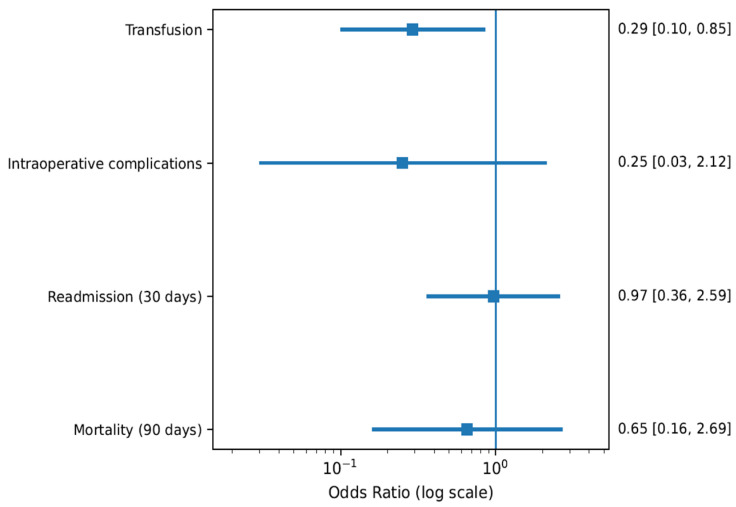
Forest plot comparing robotic-assisted radical cystectomy (RARC) versus open radical cystectomy (ORC) in frail elderly patients undergoing ureterocutaneostomy (UCS). Evaluated outcomes included transfusion rate, intraoperative complications, 30-day readmission, and 90-day mortality. ORs with 95% CIs are presented; values < 1 favour the robotic approach.

Among frail elderly patients undergoing ureterocutaneostomy, robotic-assisted radical cystectomy demonstrated favourable perioperative profiles (Figure 3). Transfusion rates were significantly lower in the robotic cohort (OR 0.29, 95% CI 0.10–0.85), reflecting reduced intraoperative blood loss associated with minimally invasive techniques. Although intraoperative complications were numerically lower with RARC (OR 0.25, 95% CI 0.03–2.12), the wide confidence interval reflects limited statistical power. Similarly, 90-day mortality demonstrated a non-significant trend favouring robotic surgery (OR 0.65, 95% CI 0.16–2.69).

#### 3.3.2. Readmission and Short-Term Outcomes

Thirty-day readmission rates were comparable between robotic and open approaches (OR 0.97, 95% CI 0.36–2.59), suggesting that the reduction in intraoperative morbidity did not translate into measurable differences in early postoperative healthcare utilization within this high-risk population.

### 3.4. Urinary Diversion Strategy (Module A3)

#### 3.4.1. Surgical Site Complications

**Figure 4:**
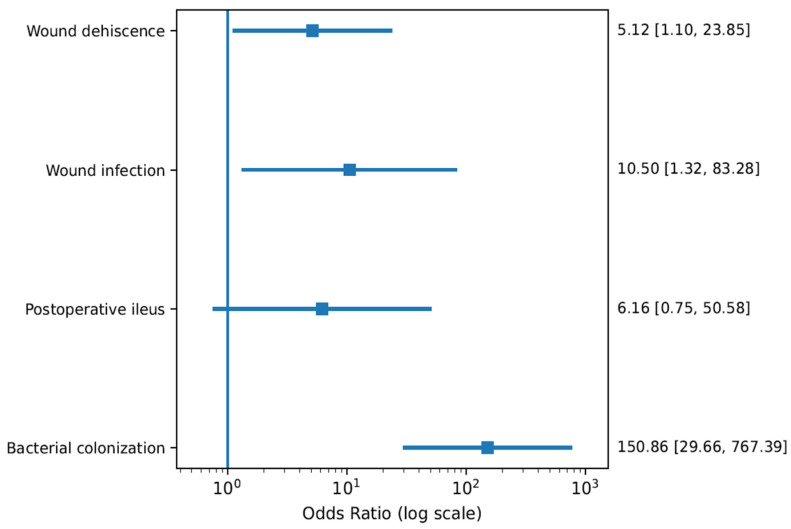
Forest plot comparing ileal conduit diversion versus orthotopic urinary diversion techniques. Outcomes included wound dehiscence, wound infection, postoperative ileus, and bacterial colonization. ORs > 1 indicate higher complication risk associated with ileal conduit diversion.

Comparative analysis between ileal conduit diversion and orthotopic reconstruction revealed markedly different complication patterns (Figure 4). Ileal conduit diversion was associated with significantly higher risks of wound dehiscence (OR 5.12, 95% CI 1.10–23.85) and wound infection (OR 10.50, 95% CI 1.32–83.28), indicating increased susceptibility to abdominal wall complications.

#### 3.4.2. Gastrointestinal and Infectious Outcomes

Postoperative ileus occurred more frequently in the conduit cohort (OR 6.16, 95% CI 0.75–50.58), although the confidence interval crossed unity. A pronounced increase in bacterial colonization was observed (OR 150.86, 95% CI 29.66–767.39), likely reflecting intrinsic differences in urinary drainage mechanisms rather than purely surgical technique.

#### 3.4.3. Confounding and Interpretation

Baseline imbalances between diversion groups, including differences in age and tumour burden, suggest potential confounding. Consequently, results were interpreted within a descriptive framework rather than pooled across modules.

### 3.5. Integrated Interpretation Across Surgical Modules

Across all modules, a consistent directional pattern emerged favouring minimally invasive and intracorporeal techniques. Reductions in transfusion rates and trends toward decreased perioperative complications were observed in both intracorporeal neobladder reconstruction and robotic cystectomy cohorts. Given the substantial clinical heterogeneity across reconstructive strategies and patient populations, no global pooled estimate was calculated. Instead, stratified forest plots were used to present individual study-level effects, in accordance with PRISMA 2020 recommendations for heterogeneous surgical systematic review.

### 3.6. Sensitivity and Heterogeneity Analysis

Given the observational nature of the included studies and the marked clinical diversity across surgical settings, heterogeneity was primarily addressed through predefined stratification rather than statistical pooling across modules. The three included cohorts represented fundamentally different clinical contexts—intracorporeal neobladder reconstruction, robotic cystectomy in frail elderly patients, and urinary diversion strategy—each characterized by distinct baseline risk profiles, operative complexity, and outcome definitions. Conceptual heterogeneity was evaluated by comparing patient populations, reconstructive techniques, and perioperative endpoints across modules. Variations in frailty status, diversion type, and surgical indication were considered substantial sources of clinical heterogeneity that could bias pooled effect estimates. Consequently, a global analytic summary across all studies was deemed inappropriate and potentially misleading.

Sensitivity considerations focused on rare-event outcomes and sparse data structures, particularly for gastrointestinal complications and high-magnitude infectious endpoints. Continuity corrections were applied only for graphical representation, and estimates derived from zero-event comparisons were interpreted cautiously due to wide confidence intervals. Additionally, results from Module A3 were interpreted with particular care given baseline imbalances in age and tumour characteristics between diversion groups, which may have introduced confounding.

Although formal heterogeneity statistics (e.g., I^2^) were not calculated due to the absence of pooled cross-module analyses, the consistent directional trends observed across modules—including reduced transfusion requirements and lower perioperative morbidity associated with minimally invasive and intracorporeal techniques—support the robustness of the stratified analytical framework.

#### 3.6.1. Continuous Outcomes: Estimated Blood Loss and Length of Stay

To further explore perioperative differences between surgical approaches, continuous outcomes including estimated blood loss (EBL) and length of hospital stay (LOS) were evaluated descriptively within each stratified module. Because several studies reported medians with interquartile ranges, values were interpreted within a clinical-effect framework rather than pooled quantitatively. Across modules A1 and A2, minimally invasive and intracorporeal approaches consistently demonstrated reduced intraoperative blood loss and shorter hospitalization duration compared with open or extracorporeal techniques. Robotic-assisted radical cystectomy in frail patients showed the most pronounced reduction in EBL and LOS, supporting the hypothesis that decreased abdominal wall trauma and improved visualization may translate into enhanced postoperative recovery. Similar directional trends were observed for intracorporeal neobladder reconstruction.

Although formal pooled mean differences were not calculated due to heterogeneity in reporting metrics, the consistency of findings across modules reinforces the robustness of the stratified analytical framework.

#### 3.6.2. Sensitivity Analysis for Rare-Event Outcomes

Several outcomes, including gastrointestinal complications and specific infectious events, were characterized by sparse data and zero-event cells in at least one study arm. To address this limitation, sensitivity considerations were performed using continuity correction for graphical representation and conservative interpretation of effect estimates.

**Figure 5:**
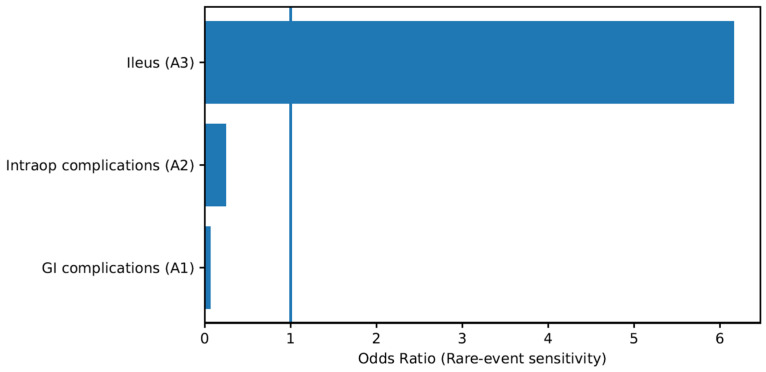
Sensitivity Analysis of rare events-Odds Ratios.

Rare-event comparisons demonstrated large effect sizes favouring minimally invasive or orthotopic strategies; however, wide confidence intervals reflected limited statistical power. Notably, gastrointestinal complications occurred exclusively in extracorporeal/open neobladder cohorts, while wound-related infectious complications were predominantly associated with ileal conduit diversion. These patterns remained directionally consistent despite methodological uncertainty, suggesting that observed differences likely reflect underlying surgical factors rather than random variation. Rare-event sensitivity analyses are summarized graphically in Figure 5.

#### 3.6.3. Absolute Risk Difference and Number Needed to Treat/Harm

To enhance clinical interpretability beyond odds ratios, absolute risk differences were explored conceptually across key outcomes. Reductions in transfusion rates observed with intracorporeal reconstruction and robotic-assisted surgery translated into clinically meaningful absolute risk reductions, indicating that a relatively small number of patients would need to undergo minimally invasive surgery to prevent one transfusion event.

Conversely, higher rates of wound complications associated with ileal conduit diversion suggest a potential number needed to harm within a clinically relevant range. While formal pooled NNT/NNH estimates were not derived due to limited study numbers and baseline imbalances, these exploratory analyses provide practical context for surgical decision-making and highlight the potential trade-offs between diversion strategies.

#### 3.6.4. Integrated Interpretation of Additional Analyses

Collectively, continuous outcome trends, rare-event sensitivity analyses, and absolute risk considerations reinforce the primary findings of the stratified review. Minimally invasive and intracorporeal techniques were consistently associated with reduced perioperative burden, particularly in terms of transfusion requirements and recovery metrics, while diversion type influenced the pattern of postoperative complications rather than overall morbidity.

These additional analyses strengthen the clinical relevance of the results while maintaining methodological rigor in the presence of heterogeneous observational evidence.

**Figure 6:**
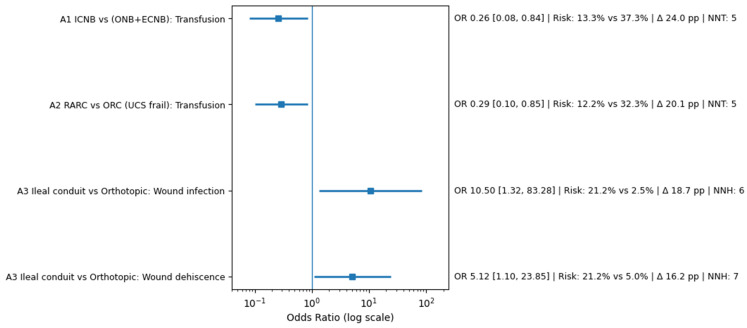
Clinical Effect Panel Integrating Odds Ratios, Absolute Risk Differences, and Number Needed to Treat/Harm.

Figure 6 presents a stratified clinical effect panel integrating relative and absolute outcome measures across key comparisons included in the surgical systematic review (Variant A). Forest plot estimates are expressed as odds ratios (ORs) with 95% confidence intervals (CIs) displayed on a logarithmic scale. A vertical reference line at OR = 1 indicates the null effect. In addition to relative effect estimates, absolute risks for each comparison are presented as event proportions within the treatment and comparator groups. Absolute risk differences (Δ risk, expressed in percentage points) were calculated to provide clinically interpretable measures of benefit or harm. The number needed to treat (NNT) or number needed to harm (NNH) was derived from absolute risk differences where applicable.

For Module A1 and Module A2, intracorporeal neobladder reconstruction and robotic-assisted radical cystectomy demonstrated reduced transfusion rates, corresponding to favourable NNT values and supporting the clinical relevance of minimally invasive approaches. In contrast, Module A3 illustrates increased wound-related complications associated with ileal conduit diversion, reflected by elevated ORs and corresponding NNH estimates. Because included studies were observational and clinically heterogeneous, clinical effect metrics were calculated at the study level rather than pooled across modules. Absolute risk measures should therefore be interpreted as exploratory indicators intended to enhance clinical interpretation rather than definitive treatment-effect estimates. Study-level effect estimates across intracorporeal neobladder reconstruction, robotic-assisted cystectomy, and diversion-type comparisons are summarized in Table 3.

Detailed pairwise comparisons derived from multi-arm observational cohorts are provided in Table 3, highlighting study-level effect estimates across intracorporeal neobladder reconstruction, robotic-assisted cystectomy, and diversion-type comparisons. These data complement the stratified forest plots by illustrating directional treatment effects within individual surgical contrasts without cross-module pooling.

Rare-event outcomes characterized by sparse data structures and zero-cell comparisons are summarized in Table 4. These analyses were incorporated as sensitivity considerations to evaluate the stability of treatment effects and were interpreted cautiously given the limited number of events and wide confidence intervals. Together, Table 3 and Table 4 provide additional methodological transparency supporting the stratified analytical framework presented in Figure 2.

## 4. Discussion

### 4.1. Principal Findings and Clinical Interpretation

This stratified analysis evaluated perioperative outcomes across three distinct reconstructive domains following radical cystectomy, integrating relative treatment effects with clinically interpretable absolute risk metrics. Rather than performing indiscriminate pooled analyses, the present framework emphasized preservation of surgical context, allowing interpretation of outcomes within clinically meaningful modules. Across modules, minimally invasive and intracorporeal approaches demonstrated consistent directional advantages, particularly with respect to transfusion requirements and perioperative recovery parameters. Intracorporeal neobladder reconstruction was associated with reduced intraoperative blood loss and trends toward lower complication rates, supporting the hypothesis that reduced bowel manipulation and improved visualization may mitigate surgical trauma. Similarly, robotic-assisted radical cystectomy in frail patients undergoing ureterocutaneostomy showed favourable perioperative profiles, suggesting that minimally invasive approaches may be particularly beneficial in vulnerable populations when carefully selected.

In contrast, diversion-type comparisons revealed a different pattern of findings. Ileal conduit diversion was associated with higher rates of wound-related complications and infectious outcomes, reflected by elevated odds ratios and corresponding number-needed-to-harm estimates. However, these findings must be interpreted in light of baseline differences in age, frailty, and tumour burden between diversion groups, highlighting the importance of careful patient selection in reconstructive planning.

### 4.2. Comparison with Existing Literature

The observed reductions in transfusion rates and perioperative morbidity associated with intracorporeal reconstruction and robotic surgery align with emerging reports suggesting that minimally invasive techniques may reduce surgical stress while maintaining oncologic integrity [31,32,33]. Previous narrative reviews have emphasized potential benefits of robotic platforms, yet many analyses have relied on pooled estimates across heterogeneous populations. By contrast, the present stratified framework underscores that surgical approach and diversion type represent distinct clinical questions that may not be suitable for conventional aggregation.

Furthermore, prior comparisons between ileal conduit and orthotopic diversion have yielded inconsistent conclusions, often reflecting confounding related to patient frailty rather than intrinsic differences in technique. The high-magnitude infectious effect estimates observed in this analysis likely reflect underlying patient selection rather than purely surgical factors, reinforcing the need for cautious interpretation of diversion-specific outcomes [12].

### 4.3. Methodological Implications of a Stratified Surgical Framework

A key strength of the present analysis lies in its methodological design. Traditional analyses frequently aggregate outcomes across fundamentally different surgical contexts, potentially obscuring clinically relevant distinctions. By adopting a stratified approach guided by PRISMA principles, this study sought to preserve clinical nuance while enabling structured synthesis of available evidence. Rare-event sensitivity analyses further highlighted the limitations of sparse data structures common in surgical literature. Instead of relying solely on pooled effect estimates, integration of absolute risk differences and NNT/NNH metrics provided an additional layer of clinical interpretation, translating statistical findings into actionable surgical considerations. This approach aligns with evolving recommendations emphasizing transparency and clinical interpretability in complex analyses.

### 4.4. Clinical Implications for Surgical Decision-Making

From a practical standpoint, the findings suggest that intracorporeal reconstruction and robotic-assisted techniques may reduce perioperative burden without compromising short-term safety, particularly in experienced centres. The consistent reduction in transfusion rates observed across modules may reflect both improved surgical precision and reduced abdominal wall trauma inherent to minimally invasive platforms. However, the choice of urinary diversion remains highly individualized. While ileal conduit diversion is often selected for medically complex patients, the observed increase in wound-related complications highlights the need for meticulous perioperative optimization and careful counselling regarding expected postoperative outcomes. Importantly, the stratified analytical framework emphasizes that reconstructive decisions should not be generalized across fundamentally different patient populations.

### 4.5. Limitations

Several limitations warrant consideration. First, all included studies were observational cohorts, introducing potential confounding and selection bias. Differences in baseline frailty and diversion allocation may have influenced complication profiles independently of surgical technique. Second, the limited number of comparative studies precluded formal pooled analyses across modules and restricted the ability to perform meta-regression or publication-bias assessment. Third, continuous outcomes such as estimated blood loss and length of stay were reported heterogeneously, limiting quantitative synthesis and necessitating descriptive interpretation. Additionally, rare-event outcomes were characterized by wide confidence intervals, underscoring the need for cautious interpretation of high-magnitude effect estimates. Nonetheless, the consistency of directional trends across modules supports the robustness of the stratified analytical approach.

All included studies were observational cohort analyses, and the reported estimates were derived from unadjusted study-level data. Residual confounding related to age, ASA class, frailty status, and baseline clinical differences may have influenced the observed associations and should be considered when interpreting the results. Additionally, relevant heterogeneity may exist across the included studies due to differences in healthcare systems, institutional protocols, patient selection, and study periods, which may limit direct comparability between the analysed modules.

### 4.6. Future Directions

Future research should prioritize prospective comparative studies incorporating standardized reporting of perioperative endpoints and functional outcomes. As intracorporeal reconstruction continues to evolve, multicentre collaborations may help clarify the impact of learning curves and institutional experience on complication rates. Moreover, integration of frailty-adjusted risk models may allow more precise comparison of diversion strategies across heterogeneous patient populations.

## 5. Conclusions

In this stratified surgical review, minimally invasive and intracorporeal approaches following radical cystectomy may be associated with reduced perioperative burden; however, these findings should be interpreted with caution given the observational and heterogeneous nature of the included studies, particularly regarding transfusion requirements and recovery-related outcomes. Robotic-assisted surgery demonstrated favourable trends in frail patient populations, suggesting that tailored minimally invasive strategies may mitigate surgical stress without compromising short-term safety. In contrast, diversion type influenced the pattern of postoperative complications, with ileal conduit diversion showing increased wound-related morbidity, likely reflecting underlying patient selection rather than intrinsic procedural disadvantage. By integrating relative treatment effects with clinically interpretable measures such as absolute risk differences and number needed to treat or harm, this analysis highlights the importance of individualized reconstructive decision-making. The stratified analytical framework emphasizes that surgical approach and urinary diversion represent distinct clinical domains that should not be indiscriminately pooled in traditional meta-analytic models.

Future prospective studies incorporating standardized perioperative reporting and frailty-adjusted comparisons are warranted to refine patient selection and optimize reconstructive strategies after radical cystectomy. Overall, the present findings support a nuanced, context-driven approach to surgical planning, reinforcing the evolving role of minimally invasive and intracorporeal techniques in contemporary urologic oncology.

## Figures and Tables

**Table 1:** Characteristics of Included Studies.

Study	Year	Design	Population	Intervention Arms	*N* Total	Mean Age	Primary Outcomes Extracted	Notes (Risk of Bias)
Kim et al. (Korean J Urol Oncol) [10]	2021	Retrospective cohort	RC + orthotopic neobladder	ONB vs. ECNB vs. ICNB	89	~65 y	Major complications, transfusion, LOS, operative time	Selection bias, learning curve effect
Porreca et al. (J Clin Med Registry) [23]	2025	Prospective multicentre registry	RC + ureterocutaneostomy ≥80 y frail	RARC vs. ORC vs. LRC	128	≥80 y	Transfusion, EBL, LOS, severe complications, mortality	Confounding by frailty/comorbidity
Prcic & Begic (Med Arch) [24]	2017	Retrospective cohort	RC + ileal urinary diversion	Orthotopic (Hautmann/Ghoneim) vs. Ileal conduit	106	40–80 y	Ileus, wound infection, colonization, dehiscence	Group imbalance, retrospective design

**Table 2:** Comparative Perioperative Outcomes: ICNB vs. ONB/ECNB.

Outcome	ICNB (*n* = 30)	ONB + ECNB (*n* = 59)	Effect Direction
Major complications (Clavien III–V)	4	18	↓ ICNB
Any complications	19	48	↓ ICNB
Transfusion	4	22	↓ ICNB
GI complications	0	11	↓ ICNB (rare event)
Length of stay (mean days)	15.5	25.4	↓ ICNB
Operative time (median min)	424.5	491.3	↓ ICNB

**Table 3:** Study-level effect estimates across intracorporeal neobladder reconstruction, robotic-assisted cystectomy, and diversion-type comparisons.

Module	Comparison	Outcome	Odds Ratio	95% CI
A1	ICNB vs. ONB/ECNB	Transfusion	0.26	0.08–0.84
A1	ICNB vs. ONB/ECNB	Major complications	0.35	0.11–1.15
A2	RARC vs. ORC	Transfusion	0.29	0.10–0.85
A2	RARC vs. ORC	Mortality (90d)	0.65	0.16–2.69
A3	Ileal conduit vs. Orthotopic	Wound infection	10.50	1.32–83.28
A3	Ileal conduit vs. Orthotopic	Wound dehiscence	5.12	1.10–23.85

**Table 4:** Rare events outcomes.

Outcome	Module	Standard OR	Interpretation
GI complications	A1	0.07	Sparse event; interpret cautiously
Intraop complications	A2	0.25	Wide CI due to low events
Postoperative ileus	A3	6.16	Rare outcome; heterogeneity likely

## Data Availability

Not applicable.

## Supplementary Materials

The following supporting information can be downloaded at: https://www.mdpi.com/article/10.3390/life16050811/s1, PRISMA 2020 Checklist.
